# Effects of adaptive servo-ventilation therapy on cardiac function and remodeling in patients with chronic heart failure (SAVIOR-C): study protocol for a randomized controlled trial

**DOI:** 10.1186/s13063-014-0530-z

**Published:** 2015-01-16

**Authors:** Yoshihiko Seino, Shin-ichi Momomura, Yasuki Kihara, Hitoshi Adachi, Yoshio Yasumura, Hiroyuki Yokoyama, for the SAVIOR-C investigators

**Affiliations:** Department of Cardiology, Cardiovascular Center, Nippon Medical School Chiba Hokusoh Hospital, 1715 Kamagari, Inzai, Chiba 270-1694 Japan; Division of Cardiovascular Medicine, Saitama Medical Center, Jichi Medical University, 1-847 Amanuma-cho, Omiya-ku, Saitama, Saitama 330-8503 Japan; Department of Cardiovascular Medicine, Hiroshima University Graduate School of Biomedical & Health Sciences, 1-2-3 Kasumi, Minami-ku, Hiroshima, Hiroshima 734-8551 Japan; Division of Cardiology, Gunma Prefectural Cardiovascular Center, 3-12 Kamiizumi-machi, Maebashi, Gunma 371-0004 Japan; Cardiovascular Division, Osaka National Hospital, 2-1-14 Hoenzaka, Chuo-ku, Osaka 540-0006 Japan; Department of Cardiovascular Medicine, National Cerebral and Cardiovascular Center, 5-7-1 Fujishirodai, Suita, Osaka 565-8565 Japan

**Keywords:** Adaptive servo-ventilation, Chronic heart failure, Noninvasive positive pressure ventilation, Left ventricular remodeling, Left ventricular ejection fraction, Cardiac function, Prognosis, Quality of life, Nonpharmacotherapy, Sleep-disordered breathing

## Abstract

**Background:**

Adaptive servo-ventilation (ASV) therapy, which is a form of noninvasive positive pressure ventilation therapy and uses an innovative ventilator that has simple operability and provides good patient adherence, potentially has therapeutic benefits—suppression of the deterioration and progression of chronic heart failure (CHF) and a reduction in the number of repeated hospitalizations. Therefore, ASV therapy draws attention as a novel, noninvasive nonpharmacotherapy for patients with CHF owing to its hemodynamics-improving effect, and it is currently being accepted in real-world clinical settings in Japan. However, clinical evidence sufficient for treatment recommendation is lacking because a multicenter, randomized, controlled study of ASV therapy has never been conducted.

**Methods/Design:**

The present study is a confirmatory, prospective, multicenter, collaborative, open-label, blinded-endpoint, parallel-group, randomized, controlled study. At 40 medical institutions in Japan, 200 Japanese outpatients with mild to severe CHF (age: ≥ 20 years; New York Heart Association classification: greater than or equal to class II) will be randomly assigned to either of the following two study groups: the ASV group, in which 100 outpatients undergo guideline-directed medical therapy and ASV therapy for 24 weeks; and the control group, in which 100 outpatients undergo only guideline-directed medical therapy for 24 weeks. The objective of the present study is to confirm whether the ASV group is superior to the control group concerning the improvement of left ventricular contractility and remodeling, both assessed by two-dimensional echocardiography. Furthermore, the present study will also secondarily examine the effects of ASV therapy on the prognosis and quality of life of patients with CHF.

**Discussion:**

ASV therapy using the device has the potential to provide therapeutic benefits based on its simple operability and good patient adherence and possesses the potential to improve left ventricular contractility and remodeling. Therefore, the present study is expected to afford more solid scientific evidence regarding ASV therapy as a novel, noninvasive, nonpharmacological, in-home, long-term ventilation therapy for patients with mild to severe CHF.

**Trial registration:**

UMIN identifier: UMIN000006549, registered on 17 October, 2011.

**Electronic supplementary material:**

The online version of this article (doi:10.1186/s13063-014-0530-z) contains supplementary material, which is available to authorized users.

## Background

In recent years, pharmacotherapy for chronic heart failure (CHF) has made remarkable progress. Furthermore, nonpharmacotherapies (for example, cardiac resynchronization therapy using an implantable cardioverter-defibrillator and comprehensive cardiac rehabilitation) have shown a given level of efficacy. However, patients with severe CHF still have a poor life prognosis and are often and repeatedly admitted to the hospital in the chronic clinical course of the disease. In addition, there are many patients whose symptoms of pulmonary congestion (for example, dyspnea and orthopnea) as well as quality of life are not sufficiently improved by existing therapeutic modalities.

Noninvasive positive pressure ventilation (NPPV) therapy has been reported to improve not only dyspnea and respiratory distress caused by cardiogenic pulmonary edema but also cardiovascular hemodynamics [1]. Namely, NPPV therapy is beneficial for both the respiratory and hemodynamic functions of patients with heart failure. In Japan, NPPV therapy is recommended for patients with acute heart failure who do not respond to oxygen therapy as a therapy categorized to class I in treatment recommendation and to A in evidence level [2]. These facts lead us to conjecture that NPPV therapy is potentially effective for patients presenting with symptoms that are attributable to the insufficient long-term management of pulmonary congestion/edema (for example, dyspnea at rest, intense fatigability, orthopnea, and paroxysmal nocturnal dyspnea). Nevertheless, NPPV therapy has not become established as a therapeutic modality for CHF because conventional medical devices for NPPV therapy are difficult to use for a long period due to their problems (for example, cumbersome operability of the devices and poor patient adherence).

Adaptive servo-ventilation (ASV) therapy, which is a form of NPPV therapy and uses an innovative ventilator AutoSet CS™ (Teijin Pharma Limited, Tokyo, Japan), potentially has the following mechanistic features that differ from conventional NPPV therapy including continuous positive airway pressure and bi-level positive airway pressure: 1) ASV therapy appropriately sustains ventilation by instantaneously detecting a decay in spontaneous ventilation of the patient and by providing support pressure in concert with his/her respiratory flow patterns; and 2) ASV therapy presents more physiological synchronization with the respiratory patterns [3]. ASV therapy potentially has therapeutic benefits—suppression of the deterioration and progression of CHF and a reduction in the number of repeated hospitalizations—based on the simple operability of the device and good patient adherence as compared with conventional NPPV therapy. Thus, it draws attention as a novel, noninvasive nonpharmacotherapy for patients with CHF owing to its hemodynamics-improving effect. Prior to the present study, we conducted a multicenter, retrospective, observational study [*S*tudy on the effects of *A*daptive servo-*V*entilation *I*n patients with chr*O*nic heart failu*R*e: *R*eal-world, multicenter, retrospective, observational study (SAVIOR-R)] for the objective of examining the real-world practical efficacy and limitations of ASV therapy for patients with CHF in Japan [4]. SAVIOR-R suggested that ASV therapy possibly improves the cardiac function and symptoms of patients with CHF, regardless of the severity of sleep-disordered breathing. Furthermore, prior single-center clinical studies indicated that ASV therapy improves left ventricular contractility and induces cardiac reverse remodeling [5], that pulmonary congestion improved by positive end-expiratory pressure suppresses sympathetic nerve activity [6-8], and that ASV therapy improves left ventricular ejection fraction (LVEF) and reduces brain natriuretic peptide levels [9] and further cardiac events (death and hospitalization) [10].

The present study is a confirmatory study of SAVIOR-R and aims to assess the effects of ASV therapy on left ventricular contractility and remodeling in patients with mild to severe CHF in a multicenter, randomized, controlled study. Left ventricular contractility and remodeling will be assessed by two-dimensional echocardiography.

## Methods/Design

### Study design and study organization

The present study (SAVIOR-C) is a confirmatory, prospective, multicenter, collaborative, open-label, blinded-endpoint, parallel-group, randomized, controlled study. The target number of patients is 200, and the study groups consist of the ASV group and the control group. The present study will last for 24 weeks, and the primary and secondary endpoints will be assessed at baseline (8 weeks prior to treatment onset through treatment onset), at week 12 of study (±4 weeks), on the day of study discontinuation, or on the day of study completion at week 24 (±4 weeks).

The Steering Committee will perform activities (for example, selection of study sites, investigators, and others, preparation and revision of the protocol and other documents, interpretation of questions about the study plan, coordination with investigators, establishment of the handling criteria for patients and data, determination of the acceptance of patients and data, interpretation of the study results, and preparation of study conclusions; Additional file 1). The Central Adjudication Committee will confirm in a blind manner the absence of any problems in measurement conditions and results of two-dimensional echocardiographic parameters, including LVEF—the primary endpoint—and in the subinvestigator’s assessment on CHF deterioration—the secondary endpoint, and will check whether adverse events and ventilator failure have developed in a biased fashion to a given group. The Study Promotion Committee will afford study support to study sites. The present study has been approved by the Ethical Review Board at each participating medical institution (Additional file 2) before its conduct in accordance with the Declaration of Helsinki. Prior to enrollment, all patients will provide written informed consent to be involved in the present study. The present trial is registered (UMIN identifier: UMIN000006549, registered on 17 October, 2011).

### Patients

**Figure 1:**
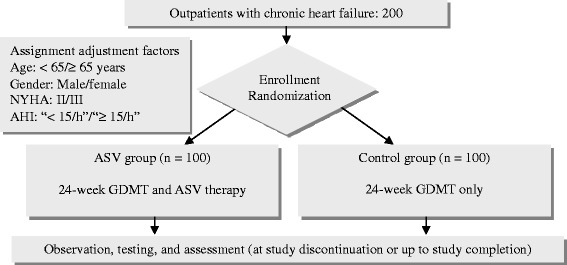
**Flow diagram of SAVIOR-C.** AHI, apnea-hypopnea index; ASV, adaptive servo-ventilation; GDMT, guideline-directed medical therapy; NYHA, New York Heart Association.

SAVIOR-C will assign 200 Japanese outpatients with CHF, who meet all of the inclusion criteria and do not fall under any of the exclusion criteria (Table 1), to either of the following two study groups at 40 medical institutions in Japan based on assignment adjustment factors [age: < 65 years/≥ 65 years: gender: male/female:, and New York Heart Association (NYHA) classification: class II/III]: the ASV group and the control group (Figure 1).

**Table 1:** **Inclusion and exclusion criteria**

**Inclusion criteria**	**Exclusion criteria**
1. Men and women	1. Patients with any of the following symptoms:
2. Age ≥20 years at informed consent acquisition	- Acute sinusitis or otitis media
3. Patients with CHF who are undergoing guideline-directed medical therapy	- Symptoms predisposing vomiting in the mask
4. Patients with CHF capable of using the ASV device at home as outpatients after enrollment	- Incapable of swallowing airway secretions
	- Pneumothorax or mediastinal emphysema
5. Patients whose NYHA class at baseline* is greater than or equal to II	- Recent cranial injury or surgery
6. Patients whose LVEF at baseline* is <40%	- Chronic hypoventilation
	2. Patients at risk of developing any of the following symptoms due to the use of the ASV device
	- Hypotension or a significant reduction in intravascular volume
	- Intense nasal bleeding leading to the risk of pulmonary aspiration
	3. Patients incapable of giving voluntary consent
	4. Patients with a history of undergoing the treatment of CHF by ASV at home
	5. Patients who are enrolled in another clinical study or trial
	6. Patients diagnosed with or suspected of dementia
	7. Patients whom the attending physician has considered ineligible for this study

*Week 8 before enrollment through day of enrollment. ASV, adaptive servo-ventilation; CHF, chronic heart failure; LVEF, left ventricular ejection fraction; NYHA, New York Heart Association.

### Study treatment

Study treatment initiates within 8 weeks after provision of the verbal and written explanations by the investigator to each patient. Eligibility check, enrollment, and randomization of each patient are performed between the provision and the study onset. Patients in the ASV group undergo ASV therapy in addition to guideline-directed medical therapy [11] for the study period (study onset through week 24 of study), while patients in the control group undergo only guideline-directed medical therapy for the study period. Patients in the ASV therapy group, who are in bed, undergo the ASV therapy each night during the study period.

### Endpoints

Efficacy, safety, and other aspects of ASV therapy will be assessed. The primary endpoint for efficacy is LVEF, a variable for the evaluation of heart failure that is applicable to a randomized controlled study of prospective, open-label, blinded-endpoint design. Two-dimensional echocardiography will be conducted at each institution in accordance with the procedures specified for SAVIOR-C. LVEF, left ventricular end-systolic volume, and left ventricular end-diastolic volume will be calculated according to the biplane Simpson’s method [12]. Furthermore, the Central Adjudication Committee will determine in a blinded fashion the acceptability of the echocardiograms to be submitted by the investigators.

The secondary endpoints for efficacy are 1) clinical events (death, hospitalization, treatment changes, and long-lasting (≥12 weeks) deterioration of symptoms) [13] to be finally adjudicated by the Central Adjudication Committee in a blind fashion, 2) brain natriuretic peptide to be measured at each participating medical institution, and 3) clinical composite response to be categorized (worsened, unchanged, and improved) based on NYHA classification and clinical events.

The endpoints for safety are adverse events and ventilator failure. The endpoints for other aspects of ASV therapy are as follows: 1) those to be collected via the case report form - patient background, quality of life, activities of daily living, vital signs, physical findings, echocardiograms, chest radiographs, hematology results, sleep study results (for example, apnea-hypopnea index), and usage of the ventilator, all from the ASV and control groups; and 2) those to be collected via electronic means—echocardiograms and air flow in the sleep study from the ASV and control groups, and usage of the ventilator from the ASV group. The schedule of observations, examinations, and assessments is shown in Table 2.

**Table 2:** **Schedule for observation, testing, and assessment**

**Acquisition means**	**Examination items**	**Timing (allowable range)**		
		Baseline: week 8 before enrollment through day of enrollment	Week 12 (±4 weeks)	Day of study discontinuation or day of study completion at week 24 (±4 weeks)
Case report form	Patient background	×		
	Combined therapy	×	×	×
	Symptoms (NYHA class and self-assessment of daytime sleepiness)	×	×	×
	Quality of life and activities of daily living	×	×	×
	Vital signs	×	×	×
	Physical examination	×	×	×
	Echocardiography	×	×	×
	Chest X-ray	×	×	×
	Brain natriuretic peptide	×	×	×
	Hematology	×	×	×
	Sleep study	×^1^	×	Wherever possible^2^
	Cardiac event		×	×
	Usage of the ventilator in the ASV group	×	×	×
	Adverse events		×	×
Electronic means	Echocardiogram	×	×	×
	Air flow in the sleep study	×^1^		Wherever possible^2^
	Usage of the ventilator in the ASV group		×	×

^1^Without the ASV device, a device for sleep study (type 1, 2, or 3) is used. ^2^Without the ASV device, a device for sleep study (types 1, 2, 3, or 4) is used. ASV, adaptive servo-ventilation; NYHA, New York Heart Association.

### Statistical methods and sample size

The subgroup analysis of patients with reduced LVEF (<40%) in SAVIOR-R revealed a 6.9% improvement (before ASV therapy: 26.5 ± 7.4%; after ASV therapy: 33.4 ± 13.0%). In SAVIOR-R, furthermore, a statistical analysis considering time-course changes in LVEF indicated that the LVEF-improving effect of ASV therapy tended to appear at week 12 of the study or later. LVEF remained unchanged when ASV therapy was not conducted and improved by 5% when ASV therapy was conducted for 24 weeks. The target number of patients in SAVIOR-C was calculated based on an assumed standard deviation of 7.4 in reference to the pre-ASV therapy value. Consequently, power exceeds 80% when a group includes not less than 90 patients. Since sufficient power is obtained when a group includes 100 patients in consideration of a presumed withdrawal rate of 10%, a total of 200 patients—100 patients per study group—were established.

The efficacy analysis set consists of the full analysis set and the per-protocol set to be defined below. The full analysis set consists of patients who initiated ASV therapy, from whom the following patients are excluded: 1) patients in the ASV group who never used the ventilator during the study period; and 2) patients from whom the efficacy data after study onset were not obtained at all during the study period. The per-protocol set consists of patients in the full analysis set, from whom the following patients are excluded: 1) patients who deviated from the inclusion criteria or fell under any of the exclusion criteria; 2) patients who fell under any of prohibited combination therapies; and 3) patients with poor compliance in ventilator use. Poor compliance is defined as an ASV therapy practice rate of < 80% (number of days from day of treatment onset through day of treatment discontinuation or day of study completion). The safety of the study groups will be analyzed statistically on an intention-to-treat basis.

The aim of the statistical analyses is to examine the efficacy and safety of ASV therapy for the target number of 200 patients by comparing the ASV group with the control group with respect to the primary endpoint for efficacy—LVEF—and to the measured values of respective secondary endpoints. The detailed procedures for randomized assignment will be determined but will not be informed to the subinvestigators at each participating medical institution. The details and technical methods of the statistical analyses will be described in the statistical analysis plan to be prepared prior to database lock. Statistical tests (for example, chi-square test using the contingency table, Fisher’s exact probability test, Kruskal-Wallis test, and analysis of variance) will be used in accordance with the scale of demographic data. Furthermore, a two-tailed probability of 15% will be established as an index of intergroup homogeneity. The data to be collected in the present study will be analyzed by the Japan-Clinical Research Support Unit that is completely independent from study organizations.

## Discussion

The 5-year survival rates of patients with CHF in the 1990s were as low as 25% and 38% for males and females, respectively [14]. The 5-year survival rate of patients with CHF who had left ventricular systolic dysfunction improved to 52% [15]. However, in both males and females, CHF requiring hospitalization was more “malignant” than many of the common types of cancer [16]. The 5-year survival rates of patients with CHF aged 65 to 74 years were 54% and 40% for males and females, respectively [17]. Furthermore, the 5-year survival rates of patients with CHF categorized to stages A, B, C, and D were 97%, 96%, 75%, and 20%, respectively [18]. These epidemiologic data clearly indicate that CHF, once progressed, still exhibits a poor life prognosis despite recent progress in therapeutic modalities and that preventing the deterioration and progression of CHF severe enough to require hospitalization is critically important for the further betterment of the survival rate of patients with CHF, and also indicates the need to develop a novel therapeutic modality which is useful for the prevention and progression of CHF, apart from pharmacotherapies and nonpharmacoptherapies that are recommended by guidelines [11,19,20].

Nonpharmacotherapies that are currently applied to the treatment of CHF include 1) cardiac resynchronization therapy using an implantable cardioverter-defibrillator to treat heart failure involving dyssynchrony of left ventricular contraction [21-23], 2) comprehensive cardiac rehabilitation including exercise therapy [24-26], 3) Waon therapy—soothing warmth therapy, a novel systemic treatment for heart failure by means of dry far-infrared radiation sauna equipment [27,28], 4) surgical approaches including coronary revascularization [29], on- or off-pump coronary artery bypass grafting [30], mitral annuloplasty [31], and mitral valve replacement [32] to treat functional mitral regurgitation caused by left ventricular volume expansion due to left ventricular remodeling, 5) auxiliary circulation including intraaortic balloon pumping [33] and extracorporeal auxiliary artificial heart [34], and 6) heart transplantation [35]. Nevertheless, none of these therapeutic modalities are practicable at home, and all of them cause great physical, mental, and economic burdens to the patient. The estimated medical cost of pharmacotherapy for the treatment of heart failure in the United States is $3.2 billion annually, and about three-quarters of the total treatment costs for heart failure are related with admissions to the hospital, in-hospital treatment, and nursing care in nursing homes [36]. Heart failure accounts for 56% of causes of death for patients with NYHA class IV, while sudden death (for example, fatal ventricular arrhythmias) accounts for 64% and 59% of causes of death for patients with NYHA classes II and III, respectively [37]. In Japan, furthermore, the percentages of patients categorized to NYHA classes I to IV at admission were 1.2%, 11.4%, 44.6%, and 42.9%, respectively [2]. Therefore, it is vitally important in the therapeutic strategy and medical economics of CHF to reasonably reduce the percentages of NYHA class III and IV patients, as well as the number of repeated admissions to hospital.

Positive end-expiratory pressure generated by NPPV therapy has the following physiologic mechanisms: alleviation of preload via a decrease in venous return [38], suppression of sympathetic overactivity [6,7], and relief of afterload via a reduction in transmural pressure [39,40]. The activation of these mechanisms is also expected during in-home therapy for the treatment of CHF. Nevertheless, conventional NPPV therapy has the limitations of cumbersome operability of the ventilator and poor patient adherence, and has not sufficiently diffused as a therapeutic modality for CHF that brings therapeutic benefits. AutoSet CS™, originally developed for the treatment of sleep-disordered breathing, demonstrates extensive improvements in the abovementioned drawbacks of the conventional medical devices for NPPV therapy. ASV therapy is being accepted rapidly among Japanese cardiologists who expect the circulatory and respiratory function-improving effects of the device. SAVIOR-R, which investigated the practice efficacy and limitations of ASV therapy for real-world patients with CHF in Japan, clearly revealed these facts and also suggested, as with other prior studies [9,10], that ASV therapy improves the cardiac function of patients with CHF, regardless of the severity of sleep-disordered breathing. The present study plans to investigate these expectations in terms of the multicenter, randomized, controlled study because of its confirmatory nature of SAVIOR-R.

ASV therapy has the potential to improve left ventricular contractility via reverse remodeling, exerting therapeutic benefits based on the simple operability of the device and good patient adherence as compared with conventional NPPV therapy, and improving the prognosis and quality of life of patients with CHF. Therefore, the present study is expected to afford more solid scientific evidence about ASV therapy as a novel, noninvasive, nonpharmacological, in-home, long-term ventilation therapy for patients with CHF.

## Trial status

The trial is ongoing.

## Additional files

Additional file 1:
**Lists of the Steering Committee, Central Adjudication Committee, Study Promotion Committee, and Advisors.**
Additional file 2:
**A list of 41 Ethical Review Boards that approved SAVIOR-C.**
Additional file 3:
**A list of investigators at the participating medical institutions of SAVIOR-C.**

## Abbreviations

ASV: adaptive servo-ventilation
CHF: chronic heart failure
LVEF: left ventricular ejection fraction
NPPV: noninvasive positive pressure ventilation
NYHA: New York Heart Association
SAVIOR-C: a *C*onfirmatory, multicenter, randomized, controlled *S*tudy of *A*daptive servo-*V*entIlation *I*n patients with chr*O*nic hea*R*t failure
SAVIOR-R: *S*tudy on the effects of *A*daptive servo-*V*entilation *I*n patients with chr*O*nic heart failu*R*e: *R*eal-world, multicenter, retrospective, observational study
