# Supplementary material for: Effects of adaptive servo-ventilation therapy on cardiac function and remodeling in patients with chronic heart failure (SAVIOR-C): study protocol for a randomized controlled trial
Source: Trials. 2015 Jan 16;16:14. doi: 10.1186/s13063-014-0530-z (PMC4331142; doi:10.1186/s13063-014-0530-z)
Supplement: Additional file 1: — Lists of the Steering Committee, Central Adjudication Committee, Study Promotion Committee, and Advisors. [file 13063_2014_530_MOESM1_ESM.docx]

Additional file 1 Lists of Steering Committee, Central Adjudication Committee,

Study Promotion Committee, and Adviser

Steering Committee: Shin-ichi Momomura (Principal Investigator, Saitama Medical Center,

Jichi Medical University), Yoshihiko Seino (Nippon Medical School Chiba Hokusoh Hospital), Yasuki Kihara (Hiroshima University), Hitoshi Adachi (Gunma Prefectural Cardiovascular Center), Yoshio Yasumura (Osaka National Hospital), and Hiroyuki Yokoyama (National Cerebral and Cardiovascular Center)

Central Adjudication Committee (Independent Data and Safety Monitoring Committee):

Kiyoshi Yoshida (Kawasaki Medical School), Akihiro Hayashida (Kawasaki Medical School), Naoki Sato (Nippon Medical School Musashi-Kosugi Hospital), Takeshi Hozumi (Osaka City University), Hiroshi Yamashita (the University of Tokyo), Kazuhiro Yamamoto (Tottori University)

Study Promotion Committee: Hidetsugu Asanoi (Imizu Municipal Hospital), Shinichi Ando

(Kyushu University), Masaaki Ito (Mie University), Hiroshi Inoue (Toyama University),

Hisao Ogawa (Kumamoto University), Masataka Sata (Tokushima University),

Hiroaki Shimokawa (Tohoku University), Hiroyuki Tsutsui (Hokkaido University),

Hitonobu Tomoike (Sakakibara Heart Institute), Tohru Masuyama (Hyogo College of Medicine), Toyoaki Murohara (Nagoya University), Tsutomu Yoshikawa (Sakakibara Heart Institute)

Adviser: Kenji Ueshima (Department of EBM Research, Institute for Advancement of Clinical and Translational Science, Kyoto University Hospital)
