# Supplementary material for: Effects of adaptive servo-ventilation therapy on cardiac function and remodeling in patients with chronic heart failure (SAVIOR-C): study protocol for a randomized controlled trial
Source: Trials. 2015 Jan 16;16:14. doi: 10.1186/s13063-014-0530-z (PMC4331142; doi:10.1186/s13063-014-0530-z)
Supplement: Additional file 2: — A list of 41 Ethical Review Boards that approved SAVIOR-C. [file 13063_2014_530_MOESM2_ESM.docx]

Additional file 2 A list of 41 Ethical Review Boards that approved SAVIOR-C

Saitama Medical Center, Jichi Medical University; Nippon Medical School Chiba Hokusoh Hospital; Hiroshima University; Gunma Prefectural Cardiovascular Center; Osaka National Hospital; National Cerebral and Cardiovascular Center; Hokkaido University Hospital; Tohoku University School of Medicine; Sakakibara Heart Institute; Toyama University; Imizu municipal hospital; Nagoya University Graduate School of Medicine; Mie University Graduate School of Medicine; Hyogo College of Medicine Hospital; Tokushima University Hospital; Kyushu University Hospital; Faculty of Life Science Kumamoto University; Nippon Medical School Hospital; Keio University; National Defense Medical College; Anjo Kosei Hospital; National Hospital Organization Nagoya Medical Center; Nagoya Ekisaikai Hospital; Higashisumiyoshi Morimoto Hospital; Kansai Rosai Hospital; Hyogo Brain and Heart Center; National Hospital Organization Higashihiroshima Medical Center; Miyoshi Central Hospital; University of Occupational and Environmental Health; University of the Ryukyus; Saiseikai Futsukaichi Hospital; Kokura Memorial Hospital; Matsuyama Red Cross Hospital; Ehime Prefectural Central Hospital; Nagano Chuo Hospital; Kitano Hospital; Saitama Medical University; Tenri Hospital; Osaka Red Cross Hospital; Juntendo University Hospital; Matsumoto Clinic*

*: Medical institution that conducted the ethical review on the support of SAVIOR-C by

Public Health Research Foundation
