# Supplementary material for: Effects of adaptive servo-ventilation therapy on cardiac function and remodeling in patients with chronic heart failure (SAVIOR-C): study protocol for a randomized controlled trial
Source: Trials. 2015 Jan 16;16:14. doi: 10.1186/s13063-014-0530-z (PMC4331142; doi:10.1186/s13063-014-0530-z)
Supplement: Additional file 3: — A list of investigators at the participating medical institutions of SAVIOR-C. [file 13063_2014_530_MOESM3_ESM.docx]

Additional file 3 A list of investigators at the participating medical institutions of SAVIOR-C

Momomura S, Saitama Medical Center, Jichi Medical University; Seino Y, Nippon Medical School Chiba Hokusoh Hospital; Kihara Y, Hiroshima University; Adachi H, Gunma Prefectural Cardiovascular Center; Yasumura Y, Osaka National Hospital; Yokoyama H, Anzai T, National Cerebral and Cardiovascular Center; Tsutsui H, Hokkaido University Hospital; Shimokawa H, Tohoku University Hospital; Yoshikawa T, Sakakibara Heart Institute; Inoue H, Toyama University Hospital; Asanoi H, Imizu Municipal Hospital; Murohara T, Nagoya University Hospital; Ito M, Mie University Hospital; Masuyama T, The Hospital of Hyogo College of Medicine; Sata M, Tokushima University Hospital; Sunagawa K, Kyushu University Hospital; Ogawa H, Kumamoto University Hospital; Mizuno K and Fukuma N, Nippon Medical School Hospital; Fukuda K, Keio University Hospital; Adachi T, National Defense Medical College Hospital; Watarai M, Anjo Kosei Hospital; Yamada T, National Hospital Organization Nagoya Medical Center; Kato T, Nagoya Ekisaikai Hospital; Sakanoue Y, Higashisumiyoshi Morimoto Hospital; Fujita M, Kansai Rosai Hospital; Taniguchi Y, Hyogo Brain and Heart Center; Yanagihara K, National Hospital Organization Higashihiroshima Medical Center; Tanaka K, Miyoshi Central Hospital; Haruki N, University of Occupational and Environmental Health; Yasu T and Ohya Y, Hospital, University of the Ryukyus; Kadokami T, Saiseikai Futsukaichi Hospital; Iwabuchi M, Ando K, Kokura Memorial Hospital; Ashihara T and Kubo T, Matsuyama Red Cross Hospital; Okayama H, Ehime Prefectural Central Hospital; Kono T, Nagano Chuo Hospital; Izumi T, Kitano Hospital; Muramatsu T, Saitama International Medical Center, Saitama Medical University; Nakagawa Y, Tenri Hospital; Inada T, Osaka Red Cross Hospital; Kasai T, Juntendo University Hospital
